# Application of platelet-rich plasma with stem cells in bone and periodontal tissue engineering

**DOI:** 10.1038/boneres.2016.36

**Published:** 2016-12-13

**Authors:** Gabriela Fernandes, Shuying Yang

**Affiliations:** 1 Department of Oral Biology, School of Dental Medicine, University at Buffalo, The State University of New York, Buffalo, NY, USA; 2 Developmental Genomics Group, New York State Center of Excellence in Bioinformatics and Life Sciences, University at Buffalo, The State University of New York, Buffalo, NY, USA; 3 Department of Anatomy & Cell Biology, School of Dental Medicine, University of Pennsylvania, Philadelphia, PA, USA

## Abstract

Presently, there is a high paucity of bone grafts in the United States and worldwide. Regenerating bone is of prime concern due to the current demand of bone grafts and the increasing number of diseases causing bone loss. Autogenous bone is the present gold standard of bone regeneration. However, disadvantages like donor site morbidity and its decreased availability limit its use. Even allografts and synthetic grafting materials have their own limitations. As certain specific stem cells can be directed to differentiate into an osteoblastic lineage in the presence of growth factors (GFs), it makes stem cells the ideal agents for bone regeneration. Furthermore, platelet-rich plasma (PRP), which can be easily isolated from whole blood, is often used for bone regeneration, wound healing and bone defect repair. When stem cells are combined with PRP in the presence of GFs, they are able to promote osteogenesis. This review provides in-depth knowledge regarding the use of stem cells and PRP *in vitro*, *in vivo* and their application in clinical studies in the future.

## Introduction

Regenerating the lost bone is of primary concern in diseases and conditions involving bone loss, such as periodontitis, tumors, fractures, and bony defects.^1^ Autogenous bone has long been held as the gold standard of bone grafting materials; however, donor site morbidity, difficulty in obtaining it, and the prolonged healing time are its limitations.^2^ In recent years, autologous bone has been administered for the regeneration of bony defects and structures.^3^ But, the risk of disease transmission and foreign body immune reaction associated with it is high.^4^ In addition, synthetic bone grafting materials have been created and produced to mimic bony structure and cellular morphology along with promoting osteoconduction;^5^ however, the primary expenses involved in fabricating and manufacturing these graft materials preclude their extensive application.^6^ Hence, it is imperative to advocate and implement newer techniques and entities in order to overcome these limitations.^7^ Bone tissue engineering is the field of medicine that involves the regeneration and replacement of the lost bony tissue and structure.^4^ Due to the increasing demand and the paucity of the presently existing bone grafts, it has now become imperative to devise novel materials that can achieve excellent regeneration as well as reduce the drawbacks of the presently existing grafting materials.^8^ It is very important to harness the potential of cellular and molecular technology in order to develop newer grafting materials and exploit its practical applications.^9–11^

A high volume of research in bone tissue engineering has been devoted to adult stem cells, which can be isolated from tissues such as a bone marrow or adipose tissue. Mesenchymal stem cells (MSCs) have been identified as the cells which adhere to plastic, lack of expression and absence of the hematopoietic and endothelial markers and their ability to differentiate into adipogenic, chondrogenic, and osteogenic lineages.^12–14^ Adult bone marrow-derived MSCs (BMSCs) have been the focus of most studies due to the inherent potential of these cells to differentiate into various cell types. In the past decade, MSCs have been employed in the regeneration of bone, especially because of its potential to differentiate into an osteogenic lineage, which is of prime importance in the process of bone growth.^15–18^ It is also known to influence the fate of other cells through the process of paracrine signaling thus providing an osteoinductive and osteoconductive environment for the differentiation of other surrounding cells in the associated region.^19^ Furthermore, it also governs the immune modulatory potential of the neighboring cells through the secretion of prostaglandins.^20^ For MSCs to develop into an osteogenic lineage, it is crucial to have the presence of a catalyst that can accelerate its differentiation and proliferative potential without affecting its cellular structure and biology.^21^ It is also important for the catalyst to be inexpensive, biocompatible, and osteoconductive in property.^22,23^

PRP (platelet-rich plasma) was first defined in 2007 as a preparation of platelets present in a small volume of plasma containing a large amount of growth factors (GFs), which is essential for bone growth and regeneration.^24^ There are more than 15 GFs present in the PRP with the primary ones consisting of platelet-derived growth factor (PDGF), Insulin-like growth factor (IGF) and Transforming growth factor-β (TGF-β) along with their isoforms.^25^ These GFs have their origin in the alpha granules of the platelets (50–80 per platelet).^26^ However, recent studies have observed not only the presence of GFs, but also the cytokines, enzymes, proteins, and fibrinolytic and anti-fibrinolytic proteins which are release upon the activation of the platelets through a mechanical or chemical pathway.^27^ The factors required for activation may include collagen, thromboxane, calcium, magnesium, serotonin, and other platelet aggregating factors.^28^ Activation leads to an immediate burst of these GFs, thereby leading to the exhaustion of all the factors within 24 h.^29^ The benefits involved in the application of PRP in the regeneration of bone involve its availability, ease of isolation, good handling and storage properties and its application in the field of bone tissue engineering.^30^ In addition, it is autologous which eliminates the risk of disease transmission and immune rejection.^31^

GFs like PDGF, TGF, and IGF are associated with bone regeneration and growth since they consists of proteins which are known to be present in the cells during wound healing and are hence known to mimic bone healing conditions.^30^ These factors are present in abundant in the platelets and can be applied with MSCs for the regeneration of bone.^32^ In the presence of these factors, the MSCs have a greater predilection toward developing into an osteogenic lineage.^33^ It may have greater applications in the regeneration of critical-sized bony defects due to its excessive osteoconductive and osteoinductive potential.

This paper briefly covers various types of stem cell sources that have been described in the scientific literatures for use in tissue engineering applications. The majority of these studies have focused on PRP and stem cells due to their high osteogenic potential and the prospect of PRP for bone tissue engineering.

## Stem cells in bone regeneration

BMSCs are the most extensively studied stem cells for bone regeneration owing to their capacity to differentiate into an osteoblastic lineage in the presence of appropriate GFs as well as regenerate bone efficiently *in vivo*.^34^ Upon isolation from the bone marrow, these stem cells are characterized. These cells are generally easy to isolate and passage.^35^ However, the low yield of BMSCs potentiates the need for extensive *in vitro* expansion, which reduces the posttranslational survival and immunomodulatory properties of BMSCs.^36^ In addition, the age and health status of the donor is of critical importance with regard to obtaining these cells. In the mid-1960’s, Friedenstein *et al*.^37,38^ reviewed a series of experiments and hypothesized that BMSCs could be co-isolated *in vitro* alongside hematopoietic cells. BMSCs are generally referred to as MSCs, however, it should be noted that bone marrow usually consists of stromal cells which do not necessarily give rise to MSCs.^39^ Nevertheless, upon isolation, the purified BMSCs are referred to as MSCs as they are governed by the 2006 criteria and exhibit both plasticity and the ability to differentiate into chondrocytes, osteocytes, and adipocytes *in vitro* and *in vivo*.^40^ In 1997, Komori *et al.*^41^ proposed that marrow stromal cells express the master osteogenic transcription factor, runt-related transcription factor 2 (*Runx2*) and furthermore, regardless of differentiation, the BMSCs retain *Runx2* expression, suggesting an ability to shift phenotype and redifferentiate into osteoblasts.

Current research in MSCs not only aims at generating novel cell therapies but also enables in the development of cell models that facilitate the learning and study of the experiments for the basis of regeneration of tissues and the mechanisms involved.^42^ MSCs are generally obtained from the bone marrow and can be studied *in vitro*, which enables the convenient study of these tissue specific cells.^43^ MSCs are being used in *in vivo* studies because they are governed by the molecules in their environment where their anabolic capacity is modulated.^44^ There are various GFs present in the PRP which bring about the signaling of various processes leading to MSC proliferation, migration, and differentiation, and the development of an environment where healing of the tissues can occur.^45^ However, the use of MSCs is limited in a clinical setting due to the need for GFs for MSC growth and expansion, MSC low yield and the inherent heterogeneity they display in their multi-differentiation potential.^46^ This heterogeneity has been reported to occur within the same single-cell-derived colony, and no matter how pure the pure colony is, it will still result in a heterogeneous population upon passaging. In fact, these challenges and benefits from addressing emergent population heterogeneity are not limited to MSCs, and they can be considered for other tissue-derived stem cells and induced pluripotent cell populations and can be overcome by sorting by label-free biophysical markers or by biophysical or other characteristics.^47^ Because of the extremely low yields of BMSC progenitors (typically 0.001%–0.01%) obtained from bone marrow aspirates, large quantities of bone marrow must be procured, which can cause additional donor site morbidity.^48^ In addition, several passages are required in order to separate the hematopoietic population and other cell types from the pure BMSCs. Furthermore, passaging of the cells can result in the decreased potential of differentiation and this may potentiate the need for additional GFs in order to increase the differentiation and growth potential.^49^ These significant limitations have hindered the translation of BMSC into a clinical setting, leading investigators to seek alternative tissue sources from which to isolate MSCs. However, clinical studies have demonstrated that even with the pure preparations of these cells, only few of these cells can differentiate into an osteoblastic lineage.^50^ Even with seemingly pure preparations of BMSC, only 1% of BMSC in the population are susceptible to osteogenesis.^51^ It is also obvious that unfractionated BMSCs expanded in culture exhibit variable functional efficacy.^52^ Zuk *et al*.^53,54^ first discovered adipose stem cells (ASCs) in 2001 and ever since then, these cells have been used in the field of tissue engineering, especially in bone regeneration. It acts an excellent substitute to MSCs in bone formation, owing to its abundant supply, ease of isolation and because it is readily and safely accessible. Although, ASCs have been explored for their potential of bone formation, they have been able to yield very low amounts of bone in several studies. This could be attributed to the fact that they contain some populations of stromal and endothelial cells that could interfere with their whole potential towards osteogenesis.^53^ However, MSCs can be purified from ASC and be identified by checking for the expression of the cell markers CD73, CD90, CD44, and CD9. Moreover, ASCs have been shown to possess tri-lineage potential and to undergo osteogenic differentiation by itself as well as when treated with ascorbic acid, bone morphogenetic protein-2, and β-glycerophosphate.^55,56^

In, *in vivo* studies, ASC have been reported to repair critical-sized calvarial defects, to promote bone formation in appendicular defects,^57^ and to induce spinal fusion in murine models and they were able to promote bone formation for craniomaxillofacial repair in a large animal canine model.^58^ And although, large quantities of ASCs are implanted *in vivo*, these cells have to be passaged several times in order to decrease its contamination with other cell types. However, this could also reduce the osteogenic potential of the cell, decrease its viability and self-renewal as well as lower its multi-potency. ASCs are known to contain and release factors such as angiopoietin-like 1, Epidermal growth factor (EGF), Fibroblast growth factor (FGF), Hepatocyte growth factor (HGF), TGF-β, and CXC chemokine ligand 12(CXCL12), as well as the immunosuppressive cytokines prostaglandin E2 (PGE2) and indole amine 2,3-dioxygenase (IDO). However, while these factors may increase the growth and differential potential, their presence could also play a role in successfully regenerating bone without triggering a lymphocyte reaction.^59^ Overall, these cells possess tremendous potential for osteogenesis in the field of bone engineering. In 2003, Shi and Gronthos successfully isolated dental pulp stem cells from third molars with the use of the antibody STRO-1. These cells were characterized as cells with a high level of clonogenicity and proliferation and the ability to generate large calcified colonies and occasional nodules.^60^ MSC purified from the pulp of deciduous teeth is termed as DMSC (dental mesenchymal stem cell). The tooth is abundant in MSCs population and depending on the area source is termed as dental pulp stem cells (DPSCs), periodontal ligament stem cells (PDLSCs), apical papilla stem cells (SCAPs), dental follicle stem cells (DFSCs), and gingival tissue stem cells (GMSCs).^61^ The greatest advantage that lies with the use of DMSCs is its ease of isolation, and that despite the small size of the tooth, they are an abundant source of these cells.^62^ Also, extraction being a routine dental procedure, the isolation of these cells does not result into inconvenience to the patient.^60^

*In vivo* studies in literature have reported promising results of the use of DMSCs in bone regeneration. The use of DMSCs has been demonstrated to repair critical-size calvarial defects in mice by promoting the formation of new bone.^63^ In canine and swine models, the local implantation of DMSCs with β-TCP and HA/TCP scaffolds has been shown to induce successful bone regeneration in critical-sized orofacial bone defects.^64^ Ravindran *et al*. reported the occurrence of robust odontogenic differentiation and proliferation of tissue with the use of DPSCs, PDLSCs, and BMSCs to collagen/chitosan scaffolds without the use of external agents. Zhang *et al*. seeded 2×10^6^ DPSCs/per silk fiber/collagen/hexafluoro-2-propanol (HFIP) scaffold and observed that they formed soft dental pulp.^65^ However, the disadvantages associated with these cells is their heterogeneity which can affect its osteogenic potential. Moreover, several investigators have also reported the limited potential of DMSCs to regenerate bone *in vivo* due to its inability to promote de novo bone formation in the form of cortical bone and not vascularized bone as noted in a human mandibular defect.^66^

## Platelet-rich plasma

PRP was discovered in 1914 by Dimond *et al.*,^67^ when it was prepared for intravenous transfusions. Around four decades ago, platelets were primarily known to be associated with the hematopoietic system, until recently, in 1974, when Ross *et al.*,^68^ while working on smooth muscle cell culture found that the addition of platelets and calcium or the extract from the platelets derived upon activation, to the serum, increased the mitogenic activity and proliferation of the cells. This led to a breakthrough in platelet research and opened new realms in this field that no longer limited the use of platelets to hemostatic cells. In 1978, PDGF was discovered by Witte *et al.*^69^ and in 1979, Kaplan *et al.*^70^ reported that PDGF resided within the alpha granules of the platelet cells. Following that, several other GFs like TGF-β was discovered by Assoian *et al*.^71^ in 1983 as well as IGF-1,by Karey *et al*.^72^ in 1989 and vascular endothelial growth factor (VEGF) by Banks *et al*.^73^ in 1998. Autologous PRP was first used in a heart surgery in 1987 by Ferrari *et al.*, and did not report of any complications with its use.^74^ Autologous fibrin gel was first introduced by Gibble and Ness in 1990,^75^ at that time, this fibrin gel lacked the presence of platelets, and the addition of platelets to this fibrin gel arrived later. With the subsequent introduction of this PRP, the advent of its administration began in the world of bone-related surgeries especially due to the fact that the GFs, which were available but expensive and had a short span with inefficient local delivery to target cells.^76–78^ Recently, studies involving the use of PRP has gained momentum considering the cost effectiveness, availability of large quantities at a time, prolonged storage and the decreased rate of rejection as it is autogenous.^79^ PRP contains various GFs.^80^ There is currently an estimated of 600 GFs present in the PRP.^81^ These GFs are primarily responsible for inducing differentiation, enhancing healing and promote tissue regeneration.^82^ This molecular pool is released upon platelet activation that is the interaction of molecules such as collagen, thrombin, platelet-activating factor, serotonin, calcium, magnesium, thromboxane A2, and adenosine di-phosphate with platelet receptors.^83^ Also, mechanical disruption of platelets causes their activation and the subsequent release of the cellular contents. When platelets are activated, there is an initial burst of GFs that is lately stabilized and maintained in a sustained release.^84^

Recently, research in the use of PRP has been gaining momentum in the field of Bone regeneration and is being employed extensively in orthopedic surgery.^79^ PRP contains various important GFs that enable the growth of bone and even though the mechanism of the bone regeneration is not well understood presently, its ease of handling and application makes it a good agent in the orthopedic field.^85^ In addition, it reduces inflammation, provides excellent tissue healing and being autologous reduces the risk of disease transmission and immunogenic reactions.^81^

**Figure 1:**
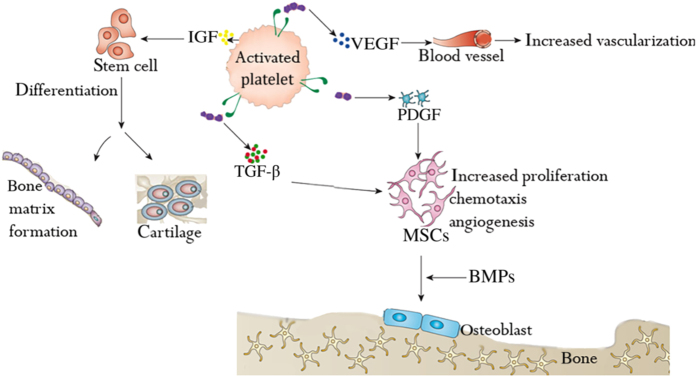
Role of PRP on stem cells in bone and cartilage formation.

When integrated with biological or synthetic bone grafts, PRP has been reported to improve the aggregation and cohesiveness of particulate-based bone substitutes, thus helping bone formation or bone regeneration process, particularly due to the fact that most of the events involved in the formation of bone are triggered by the factors that are present in PRP.^86^ However, the doubt governing whether PRP is clinically effective or not is exacerbated by the inadequate knowledge concerning the bioavailability of PRP-derived GFs.^87^ The polypeptide GFs PDGF, TGF-β, IGF-1, VEGF, HGF, EGF, and FGF have each been identified within platelet alpha granules. Many of these factors share some common structural features and signaling mechanisms, which have been summarized in Table 1 and their actions on stem cells in Figure 1.

## GFs in PRP

### Platelet-derived growth factor

PDGF was one of the earliest and first GFs to be discovered and its role and function is known to be primarily associated with all forms of wound healing by increasing the number of MSCs in the environment, by acting as a chemoattractant and recruiter of osteoprogenitor cells and by increasing the proliferation of cells at high level.^99^ PDGF has isoforms and exists as PDGFAA, PDGFBB, PDGFAB and PDGFCC. But the specific actions of each isoforms has yet to be determined.^100^ It works by binding to the transmembrane receptors.^101^ It is responsible for the mitogenesis of MSCs, endothelial mitoses into functioning capillaries, and macrophage activation for bone regeneration and wound healing.^99^ PDGFBB at amounts of 10-100 ng·mL^−1^ increased [^3^H] thymidine incorporation into the DNA and increased collagen and non-collagen synthesis in rat calvarias.^102,103^ PDGF can also increase bone collagen degradation, which is possibly related to its ability to increase collagenase production. PDGF is also known to stimulate production of several matrix molecules like fibronectin, collagen and hyaluronic acid.^104^ It is also known to stimulate the contraction of collagen matrices *in vitro*.^105^ It acts at the site of wound healing since it is released from macrophages, endothelial cells smooth muscle cells and fibroblasts cells. In some studies, it has been documented that the application of PDGF to the site of healing increased the glycosaminogycans, neovascularization and amount of granulation tissue, which implicates its role in the increase in the rate of wound healing and bone formation.^105^

### Transforming growth factor-β

TGF-β is the second most prominent GF in PRP and is responsible for the cell differentiation and proliferation.^106^ TGF-β is secreted in a latent form that is activated outside of the cell.^107^ It acts by binding to heteromeric serine/threonine kinase receptors on the cell surface that transmit a signal to nucleus via Smad proteins to ultimately regulate changes in gene expression.^107^ At normal serum levels, TGF-β can promote mitogenesis and osteogenic differentiation of osteoprogenitor cells. However at altered levels, it can cause pathological conditions in bone.^108^ Ideally, in the platelets, TGF-β is secreted in a dormant form.^109^ It is then later activated via certain proteolytic or non-proteolytic pathways. It can regulate changes in the gene expression via Smad pathway, as well as inhibit mitogenesis and cell growth through the MAP kinase and cyclin dependent pathways. TGF-β can also regulate proliferation, differentiation, alteration in cell morphology, chemotaxis and adhesion of the osteogenic progenitor cells at the site of wound healing, which makes it an excellent agent in the promotion of fracture healing.^110^

### IGF

IGF-1 is produced by osteoblasts that are generally controlled by the growth hormone and other factors. Their activity is regulated at several levels.^111,112^ They can enhance collagen synthesis as well as osteoblast differentiation and proliferation.^113^ The differentiation is dose and time dependent and functions through various signal transduction systems.^114^ With aging, there is an impaired osteoblastic function with reduced bone formation. IGF-1 has an essential role in the development of the growing skeleton and in the maintenance of bone mass during late adulthood and aging.^115^ Its mitogenic action selectively promotes cell multiplication in young differentiated clones.^116^ It can also stimulate tissue growth and has an important role in bone mass maintenance and acquisition.^116^ When administered with growth hormone, they have synergistic effect on bone modeling and regeneration.^114^

### PRP isolation, activation, and delivery

PRP is obtained by centrifuging peripheral blood in order to obtain plasma, platelets and red blood cells. The plasma is abundant in cytokines, thrombin, and other GFs, with inherent biological and adhesive properties. PRP was first prepared in the 1970s.^117^ Several papers have later modified the protocols for PRP extraction by creating changes in the time, speed and distance, with reference to centrifugation physics and mechanics with gravity in the preparation and quantity of PRP.^118–121^ The process of isolation of PRP involves the use of anti-coagulation. Do Amaral *et al*.^122^ found that there was no difference between the use of EDTA, ACD (acid citrate dextrose) and sodium citrate as an anticoagulant; however, EDTA yielded higher platelet quantity, PRP obtained by citrate solution resulted in higher induction of MSC proliferation and smallest variation in *MSC* gene expression and ACD caused an increase in platelet recovery. There are two widely used protocols in the preparation of PRP: one step and two step PRP preparation. The effect of separation by these two methods is still controversial. The increase in commercial applications led to the development of PRP kits.^123–125^ However, GFs content has been established to be the same using the presently available methods of isolation. GFs can be released from the PRP upon activation via endogenous or exogenous pathway. Endogenous activation involves using freeze thaw cycles. However, the freeze thaw cycles would affect the release of the GFs due to destruction of the platelets. Another common method of activation is the use of calcium chloride and thrombin.^126^ GF release has shown no differences when activated with either calcium chloride or thrombin. Calcium chloride is preferred over thrombin due to thrombin’s ability to act as an anticoagulant and thereby precipitate undesired reactions.^127^ Furthermore, there have controversies over the use of leukocyte rich PRP and pure PRP. Yin *et al.*^128^ reported that Leukocytes in L-PRP may activate the NF-κB pathway via the increased pro-inflammatory cytokines to induce the inferior effects on bone regeneration of L-PRP compared with P-PRP, thus implying that pure PRP may be a safer option for use.

Upon activation, PRP can release several GFs that are essential for healing and bone regeneration.^117^ These GFs have a short life span of 24 h and hence, potentiate a need for delivering PRP via different biological materials that have lower degradation rates in order to prolong its effect on bone regeneration and healing. Several studies have defined the use of platelet gels (gel formed upon activation of PRP using calcium chloride and thrombin), certain salts like calcium phosphate, calcium sulfate, nano calcium sulfate, hydrogels-like gelatin, and alginate, nanofiber scaffolds and bioactive glass.^118,129–131^ The studies have been summarized in Table 2.

## Bone regeneration process

Bone growth is a combination of two pathways, that is, intramembranous and endochondral.^157^ After the condensation and merger of the MSCs has occurred, it serves as an architecture for the process of bone formation.^158^ Intramembranous bone formation consists of the process where the mesenchymal progenitor cells differentiate into osteoblastic cells and form the mandible, clavicle and other bones.^159^ Whereas, the process of endochondral bone formation involves the process of formation of most of the long bones in the body which is characterized by the differentiation of the mesenchymal progenitor cells into chondrocytes, thereby leading to the formation of a template made of cartilage which is later replaced by bone.^160^

The process of endochondral and intramembranous bone formation is regulated by various genes and proteins that are similar even though there are several differences in the structure and composition of the bone at various levels. Several proteins like the Indian Hedgehog (Ihh), parathyroid hormone related peptide (PTHrP), BMPs, and FGF are key regulators in both the processes of bone formation.^161^ However, the BMPs, Ihh, and PTHrP are extremely essential and are required for the co-ordination of the balance between chondrocyte proliferation and hypertrophy and regulate the thickness of the growth plate.^162^ As far as the intramembranous bone formation process is concerned, these key regulator proteins stimulate the undifferentiated mesenchymal progenitor cells to differentiate into pre-osteoblastic cells which co-express chondrocytic and osteoblastic markers simultaneously.^163^

Generally, the process of bone defect repair is similar to the process of intramembranous and endochondral bone formation which occurs by the initial development of a hematoma, accompanied by an inflammatory response and the combination of several cell signaling and key regulator protein molecules like ILs, TNF-α, FGF, BMPs, PDGF, and VEGF. Interestingly, the entire process is completed without the formation of scar tissue.^164^ The healing of the bony defect at the periosteum follows the process of intramembranous bone formation which occurs because of the role of the proteins on the mesenchymal progenitor cells, whereas the soft tissues stabilize the callus by the process of endochondral bone formation which involves the transition of the totipotent MSCs into chondrocytes, followed by maturation and mineralization, leading subsequently to the process of new bone formation and subsequent remodeling of the newly formed bone.^165^

Hence, it is imperative to develop techniques to combat and mimic the bone development process or the bone defect repair process. It is indeed necessary to place importance on both the processes since both the process are nearly similar and consists of an initial hematoma formation as well as the inflammatory response to healing and both the processes require the presence of the bone GFs and signaling molecules.^166,167^ The field of bone engineering should lay emphasis on the following factors:

1. Presence of totipotent stem cells
2. Signaling pathways, genes, and proteins
3. The presence of a good scaffold that is osteoinductive and osteoconductive
4. The understanding that normal tissue healing involves progressive remodeling and restructuring of pre-existing tissue structures
5. Angiogenesis and neo vascularization

The use of PRP in bone regeneration and cartilage formation have long been implicated.^168^ Marx had proposed the first protocol for the isolation of PRP using calcium and thrombin.^29^ Additional protocols have been proposed ever since which differ in the pathway of platelet activation and leukocyte concentration.^120^ It has been noted that the absence of leukocytes may prevent the release of pro-inflammatory cytokines which might increase the inflammation during wound healing thus decreasing and interfering with the mechanism of bone growth. However, Choukroun’s technique (L-PRF) has mentioned that the presence of leukocytes in the PRP enhance the host immune defense objective and role. Also, the enzyme, myeloperoxidase contained in the monocytes and neutrophils exhibit bactericidal properties that can decrease bacterial infections and contaminations associated with the wound healing process.^118^

PRP is activated using calcium and thrombin. The addition of calcium and thrombin activates the coagulation pathway to release the GFs.^125^ Thrombin is known to contain bovine factor V and its systemic use is associated with increased blood clotting and coagulopathies leading to cross reactivity between anti-bovine factor V antibodies with human factor V.^169^ However, studies have noted that the presence of thrombin in the circulatory system may lead to the formation of blood clots thereby provoking a myocardial infarction. Therefore, it is necessary to eliminate thrombin and activate the PRP using only calcium chloride. Studies have demonstrated that there is no difference in the concentration of GFs or the degree of activation when calcium chloride is used as an activator for the coagulation pathway as compared to the use of thrombin.^132^ However, the only disadvantage to using calcium chloride in the activation of PRP involves an increased period required for activation. A thrombin receptor agonist peptide (TRAP) can also be used in lieu of the thrombin which is similar in function. It has been noted that for *in vitro* studies, it is imperative to activate the PRP in order to release the GFs embedded in it.^170^ However, *in vivo* studies may not require the activation of the PRP.^171^

When PRP is employed in conjunction with MSCs *in vitro*, the findings have demonstrated that it tends to increase the cellular proliferation but decreases the osteogenic differentiation.^172^ PRP is generally seen to have suppressed the alkaline phosphatase activity *in vitro*, however increasing the cellular proliferative activity.^173^ The increased cellular proliferative activity may result in the altered morphology of the cell distribution result in the formation of pre-osteoblastic cells.^174^ BMP2 increases the osteogenic differentiation of MSCs and appears to have a role opposite to PRP, since *in vitro* studies have not shown an increased proliferative activity of MSCs with the use of BMP2 only.^175^ When BMP was used in conjunction with PRP, PRP could inhibit the activity of BMP, thus increasing the proliferation activity but decreasing the osteogenic potential of the MSCs.^174^ After the complete release of the GFs, the MSCs regains its responsiveness to the BMPs^176^ (Table 3).

A concentration of 2%–5% PRP is ideal for the osteogenic differentiation of the MSCs *in vitro*. Any concentration below or above inhibits the osteogenic potential of MSCs.^191^ Likewise, an increase in the concentration of MSCs increases the cell proliferative activity of the MSCs. However, conditions need to be optimized for clinical studies. L-PRF can stimulate the osteogenic differentiation and proliferation of the human MSCs *in vitro* in a dose-dependent manner as compared to PRP without leukocytes.^192^ When cultured with one PRF membrane, around 160%–210% proliferation rate can be expected in comparison to the use of two membranes,where 190%–380% can be achieved for up to 14 days in culture.^184,193^

MSCs, adipose- and muscle-derived stem cells (MDSCs) and human subchondral bone-derived progenitor cells are among the commonly employed stem cells in the field of cartilage engineering.^194^ Studies have reported that PRP can induce mitogenic effects on MSCs. And when MSCs were treated with PRP *in vitro* for 7 days, while both chondrogenic and osteogenic gene markers were upregulated and the chondrogenic markers, including Sox9 and aggrecan, increased much more (over 10-fold increase) than RUNX2 (less than 2-fold increase),^195^ a marker of early osteogenic differentiation. Furthermore, when muscle-derived MSCs were treated with PRP *in vitro*, their ability to proliferate, adhere and migrate was significantly promoted, however, the chondrogenic gene expression was not upregulated.^188^ Moreover, PRP could also stimulate the vertical migration of subchondral progenitors and cartilaginous matrix accumulation *in vitro*.^196^ These reports suggest that PRP might be able to enhance the migration of the subchondral progenitors to repair cartilage defects.

Bone tissue engineering consists of a combination of cell, GFs and a good scaffold that exhibits osteoinductive and osteoconductive properties. PRP was able to modify the properties of MSCs when seeded on a synthetic scaffold, owing to the fact that the GFs present in the PRP could provide a nutritive environment to the MSCs.^197^ It was noticed that the MSCs could adhere better to the scaffold in the presence of the PRP. However, the excellent adherence was limited to the high specific surface area of the calcium scaffold in comparison to the lower surface area counterparts of β-tricalcium phosphate. In the presence of hydroxyapatite and silica coated hydroxyapatite scaffolds, platelet poor plasma was able to stimulate the osteogenic differentiation of goat MSCs greater than the platelet lysates.^198^

MSCs can also be administered in conjunction with other bone grafting materials in order to increase its bone growth potential. Demineralized bone grafts can accelerate the cellular proliferative activity of human MSCs *in vitro*, but can alsodecrease osteogenic differentiation potential.^199^ On the contrary, when human MSCs are administered alongside freeze-dried bone allografts, they can significantly increase the osteogenic differentiation potential *in vitro* in an osteogenic media along with the GFs after 1–15 days.^200,201^

There are very few methods to evaluate the conformation of the conversion of MSCs into osteoblastic cells *in vivo*. Hence, Freidenstein proposed the first *in vivo* assay for the determination of the transition of MSCs into an osteoblastic lineage and used ‘diffusion chambers’ to study the differentiation potential of MSCs.^202,203^ The gold standard assay at present to study the differentiation potential of MSCs *in vivo* is implanting MSCs into a β-tricalcium phosphate scaffold in SCID mice by subcutaneous implantation since the heterogeneity of MSC cultures and considerable donor variability preclude standardized production of MSC and point on functional deficits for some human MSC populations, this assay was developed in order to predict the therapeutic capacity of human MSC before clinical transplantation.^203,204^

In critical-sized defects, MSCs combined with PRP and BMP2 have demonstrated the greatest bone formation effect.^84^ It has been observed that a single component alone cannot achieve good results as compared to the combination of MSCs BMP2 and PRP. The only problem is the optimizing the ratio of PRP to bone graft. PRP has been shown to promote the osteogenic differentiation of osteoblastic precursors. However, when implanted *in vivo*, it does not show to have a greater effect on the regeneration process of bone as compared to the use of MSCs alone. When implanted at an ectopic region in goats, MSCs with PRP did not seem to have any significant regenerative effect on the critical-sized defects as compared to MSCs alone when MSCs was seeded on scaffolds made of hydroxyapatite and β-tricalcium phosphate.^191,205^

In bone regeneration around the dental implants in the canine mandible, it was observed that the combination of MSCs with PRP led to excellent bone formation and vascularization which was comparable to bone regenerated by autologous bone alone.^191^ When MSC was combined with PRP fibrin gel and was used for alveolar augmentation in a canine model, the findings demonstrated the highest amount of dental osteointegration. A combination of MSCs, BMP2 and PRP in a gelatin, β–tricalcium phosphate scaffold demonstrated good regeneration in an equine cartilage defect repair within 16 weeks.^206^ PRP can thus, promote bone regeneration in the beginning stages of bone formation and bone healing. However, it does not seem to have an effect on the later stages on bone formation due to early dissolution of fibrin and GFs. PRP can also effectively increase bone formation when administered in a natural or synthetic scaffold like hydroxyapatite and β-tricalcium phosphate. But, the lack in the consistency of the results can be attributed to the heterogeneity of the studies (Table 4).

## Clinical applications of stem cells and PRP in orthopedics

PRP is generally and extensively employed in the field of orthopedics for the regeneration of defects and fractures. PRP in conjunction with MSCs has been proposed for the treatment of unions of fractures, distraction osteogenesis, spinal fusions, and periodontal defects, post extraction bone regeneration and dental osteointegration (Table 5).The major advantage for using PRP is that PRP has antimicrobial effects *in vitro*. Low volume WBC containing PRP can reduce inflammation. No side effects have been reported in 146 patients that were treated with PRP for bone regeneration. Many studies have suggested safety, increased bone formation, wound healing and vascularization with the use of PRP; adverse effects are rare risks, and benefits have been thoroughly reviewed. Due to the early dissolution of PRP and fibrin resorption, the risk of developing an adverse reaction to PRP is rare.

Sinclair *et al.*^237^ in their systematic review paper described the effects of MSCs and PRP in fractures and non-unions, which included 39 papers involving trials on the effects of GFs and MSCs on non-unions or bone repair. They reported that MSCs and PRP when used as a single entity or combined together, significantly promoted fracture and non-union repair as well as synergistically enhance wound healing. When administered in conjunction, MSC and PRP demonstrated excellent implant success in titanium implants.^11^ Implants of β-TCP seeded with MSC and PRP for maxillary sinus floor augmentation were clinically stable 12 months after loading.^238^ In a randomized controlled study, it was noticed that PRP, if combined with MSC, increased the osteogenic potential of lyophilized bone chips.^239^ The strength of this clinical research was the randomization and the comparison among different treatments (freeze-dried bone allograft alone or in combination with PRP with or without MSC); its weakness was the lack of the evaluation of PRP effect in alone and of a clear primary outcome. However, more well designed protocols and studies and rigorous clinical trials using consistent techniques may lead to more conclusive general recommendations. Patients, suffering from achondroplasia (ACH), hypochondroplasia (HCH) or congenital pseudo arthrosis or distraction osteogenesis were given MSCs with PRP.^240^ The femoral lengthening showed significantly faster healing than did the tibial lengthening and transplantation of BMSCs and the PRP shortened the treatment period by accelerating new bone regeneration during distraction osteogenesis of the lower extremity in patients with ACH and HCH.^240^

## Challenges

Although several reports have elaborated on the advantages of PRP in the fields of periodontal, bone and cartilage regeneration, there are still several challenges to be addressed.^241^ To begin with, we have not yet been able to obtain consistent results from the insufficient pre-clinical and clinical data on the immunogenicity and protective effects of stem cells.^242^ Also, several questions need to be answered before these procedures can be clinically translated.^243^ First, it is important to determine the molecular immune mechanisms and cells that are involved in these responses, the dynamic fate of these implanted ASCs, assessment of its clinical efficiency and factors that influence these inconsistent results.^244^

Another challenge is in obtaining a homogenous composition of PRP, since its composition displays relatively strong intra-patient variation.^245^ Moreover, its variation is correlated with age, sex, and patient comorbidities.^246^ Furthermore, it remains unclear about how these changes will affect stem cell behavior *in vivo*.^247^ Even the preparation methodology can significantly affect its composition and there is no standard protocol in literatures to obtain PRP gel or PRP for clinical application. These incongruences reduce the comparability and reproducibility of the various PRP studies.^214^ These challenges can be addressed by initiating systematic high throughput analyses.

Stem cells like MSCs, ASCs and MDSCs have shown great promise in the field of tissue and bone engineering for the regeneration of bone, periodontium and cartilage formation.^248^ However, one of the greatest challenge is obtaining a population with negligible heterogeneity, which can be overcome by cell sorting according to its characteristics, cell markers.^249^ However, large scale production of the stem cells may be inconvenient owing to the difficulty in obtaining a population of cells that are phenotypically and genetically similar. Moreover, the effectiveness of genetically modified MSCs has been reported in different disease models.^250^ However, the stability of the transfected cell lines may appear to be a challenge and potentiates further investigation.

## Summary and perspective

**Figure 2:**
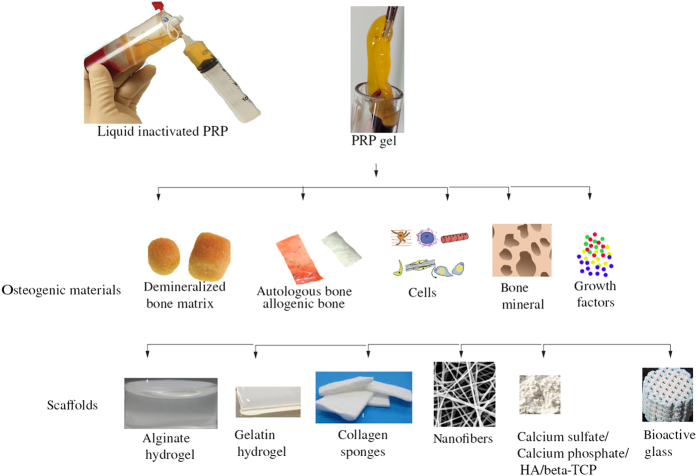
Clinical delivery of PRP for bone regeneration.

PRP consists of a cocktail of GFs which can promote osteogenic differentiation extensively and efficiently and yet appears to be inefficient as perceived from the findings in the clinical studies.^125^ The reason can be attributed to the fact that although, PRP is abundant in the bone GFs, these factors are released almost immediately when implanted into the body with the overall exhaustion of the factors occurring within twenty-four hours. The process of bone regeneration as previously described occurs over a span of 4-6 weeks. Hence, the effect of the PRP is almost negligible as far as bone healing is regarded. This makes it imperative to deliver the PRP in such a manner that the GFs may able to be sustained over the period of bone regeneration. Studies have employed the use of beads and capsules for the delivery of the PRP^147^ (Figure 2). Alginate capsules have however, demonstrated a consistent and greater concentration of GFs as compared to the beads, probably due to the chemical composition of the beads which allows the calcium ions to bind and integrate with the PRP, hence decreasing its ability to release the GFs efficiently.^251^ Moreover, the beads can be optimized to overcome this problem. The beads and capsules can also be allowed to incorporate a combination of PRP and GFs that may be able to increase the osteogenic differentiation of MSCs (unpublished data). As PRP is a good cell proliferator and BMPs are good osteogenic differentiators, it makes it feasible to administer them in conjunction with each other in order to increase the formation of bone in critical-sized defects.^252^ Furthermore, PRP with the GF beads can be double encapsulated with cements like Nano calcium sulfate. The slow degradation rate of Nano calcium sulfate would enable the PRP to be released at the peak of bone formation thus increasing its efficiency for bone formation greatly in clinical studies.^253^ As reviewed here, PRP and stem cells can be employed in conjunction for the regeneration of bone. However, since PRP leads to the cellular proliferation of pluripotent cells, it is important to incorporate a GF component as well, especially as bone Morphogenic protein since it can increase the osteogenic differentiation of bone. But, the fast degradation rate of the fibrin and the dissolution of PRP restricts its use to just in the early stages of bone healing and is negligible in the late stages of bone development. Hence, it is necessary to use and deliver the PRP via a carrier which can degrade slowly, so as to release the PRP GFs in a sustained manner. Future studies would have to be focused on developing a carrier for the delivery of GFs to increase its overall efficacy.

## Figures and Tables

**Table 1:** GFs released from PRP

Growth factor	Source	Target	Function	Reference
Platelet-derived growth factor	Platelet, macrophages, monocytes, smooth muscle, degranulating platelets, endothelial cells, macrophages, osteoblasts, osteoclasts, mesenchymal stem cells.	Smooth muscle, fibroblasts, glial cell	Cell proliferation, neutrophil chemotaxis, inducing cells to migrate toward the wound, collagen production, blood vessel repair and regeneration, connective tissue healing, Increases mitogenesis in smooth muscle cells/glial cells/fibroblasts,angiogenesis and macrophage activation, Differentiation of fibroblasts, collagenase secretion	^88^
Transforming growth factor-beta	Platelet, T-lymphocyte, macrophage, neutrophil, osteoblasts, macrophages, platelets, bone matrix	Fibroblast, stem cell, endothelial cell, epithelial cell, pre-osteoblast,	Stimulation/inhibition of endothelial, osteoblastic and fibroblastic chemotaxis and angiogenesis, collagenase secretion, mitogenesis of other GFs, increase fibroblast and osteoblast mitosis, promotion of wound healing, bone matrix formation, growth and neurogenesis of epithelial cells, Regulation of the balance between fibrosis and myocyte regeneration, stimulation of proliferation of undifferentiated mesenchymal stem cells, inhibition of replication of most cells *in vitro*, inhibition of MMP production.	^89^
Insulin-like growth factor	Osteoblasts, monocytes, chondrocyte, Macrophage, Plasma stored in bone, epithelial cells, endothelial cells, smooth muscle cells,liver	Osteoblasts, fibroblast,chondrocyte	Bone matrix formation, increase cartilage growth, wound healing, proliferation of osteoprogenitor cells, mitogenic for osteoblasts, fibroblasts and endothelial cells,	^90^
Vascular endothelial growth factor	Platelet	Endothelial cells	Angiogenesis and increased capillary permeability, expression increased in presence of hypoxia,	^91^
Epidermal growth factor	Submaxillary gland, brunner’s gland. Macrophage and platelets.	Epidermal cells,	Epithelial cell proliferation, angiogenesis and promotion of wound healing, induction of basal membrane formation, keratinocyte migration and granulation tissue formation.	^92^
Fibroblast growth factor	Mast cells,Macrophage, osteoblasts and immature chondrocytes.	Fibroblasts, endothelial cells	Cell growth, tissue repair, collagen production, hyaluronic acid production	^93^
Keratinocyte growth factor	Platelets	keratinocytes	Proliferation, differentiation and regeneration of keratinocytes	^94^
Connective tissue growth factor	Platelets	vascular endothelial cells, epithelial cells, neuronal cells, vascular smooth muscle cells, and cells of supportive skeletal tissues	Increased wound healing, angiogenesis, chondrogenesis, osteogenesis, tissue repair fibrosis.	^95^
Interleukin-8	Macrophages, epithelial cells, endothelial cells, smooth muscle cells,keratinocytes	Neutrophil, endothelial cells,macrophages, mast cells, and keratinocytes	Induction of chemotaxis in target cells and stimulate their migration toward site of infection, pro-inflammatory, recruiting fibroblasts and endothelial cells	^96^
Platelet derived angiogenesis growth factor	Platelet, endothelial cells	Endothelial cells	Angiogenesis, increased capillary permeability, stimulation of endothelial cells mitogenesis	^97^
Platelet factor-4	Platelet	Neutrophil, fibroblasts	Chemotaxis of neutrophils and fibroblasts, potent anti-heparin agent	^98^

Abbrevations: GF, growth factor; PRP, platelet-rich plasma.

**Table 2:** Delivery of PRP for bone formation

Type of material	Animal model	Results	References
Activated PRP	Goat trabecular bone implants	Increased bone-to-implant surface contact.	^132–134^
Activated PRP with BMMSCs, porous hydroxyapatite (HA),	Canine trabeculae implants	Increased bone healing	^135^
Activated PRP with HA/collagen type I bead matrix within a polytetrafluoroethylene (PTFE)	Rabbit iliac crests	Increased mineralization	^136^
Activated PRP with DBM (demineralized bone matrix)	Athymic rats	Increased osteoconductivity	^137^
PRP gel	Diabetic fracture healing rats	Increased healing	^138–140^
PRP gel	50-patient randomized clinical study for autograft positioning for ACL surgery	Increased cortical bone formation	^141,142^
PRP gel	Non-union long-bone regeneration	Lesser complications	^143^
PRP gel with autologous and/or allogeneic bone	Tibia fracture of a diabetic patient	Bridged bone defect	^144^
PRP gel with autologous and/or allogeneic bone	Rabbit tibia	Increased bone regeneration	^145^
Alginate hydrogel	MSCs *in vitro*	Increased alp and mineralization activity	^146^
Alginate beads and capsules	SaOS-2 osteoblast-like cell *in vitro*	Increased alp and proliferation	^147^
Alginate hydrogel	Mouse subcutaneous	Increased ectopic bone regeneration	^148^
Gelatin hydrogel/sponge	Rabbit ulna and calvarial defects	Increased bone regeneration	^149,150^
SEW2871, of a sphignosine-1 phosphate agonist and PRP were combined in micelles and incorporated into gelatin hydrogels	Rat	Increased recruited macrophages	^151^
Collagen sponges	Pig model with a critical-size defect (10×8 mm)	Increased bone regeneration	^152^
Biphasic calcium phosphate or deproteinized bovine bone	20 patients with sinus augmentation	Increased osseointegration	^153^
Allogenic PRP+bone morphogenic protein 7 (BMP7)+CaP coated electrospun PCL	Full thickness diaphyseal segmental rat femoral defects	Significantly increased bone volume and biomechanical properties	^154^
Bioactive glass	Premolar defects in dogs	Improved bone formation	^155^
BMSCs+PRP	Intervertebral disc degeneration in rabbits	Increased regeneration of early degenerated discs	^156^

Abbreviations: BMSC, bone marrow-derived mesenchymal stem cell; PRP, platelet-rich plasma.

**Table 3:** PRP application *in vitro*

Cell type	Application to culture	Outcome	Reference
Bone			
Mouse BMSCs	PRP-BMP2 genetically modified MSCs(2.5%–5% PRP for *in vitro* and 10% for *ex vivo*)	Improve BMSCs proliferation and differentiation	^177^
Synovial fluid MSCs	SF-MSCs-alginate system (20%–50% PRP) 50% demonstrated highest activity	Promoted MSCs proliferation and chondrocyte differentiation	^178^
MSCs derived from canine umbilical cord	Co-cultured with PRP and demineralized bone matrix.	Induced osteogenesis *in vitro* of MSCs derived from the umbilical cord	^179^
BMSCs	BMSCs+PRP	Increased MSCs proliferation and differentiation	^176^
Human ASCs	Combination of chitosan-PRP and either Nano-hydroxyapatite or tricalcium phosphate	Enhanced MSCs proliferation and differentiation	^180^
Human DPSCs	PRP (1%, 5%, 10%, 20%)	1%–10% PRP showed significant effect of promoting hDPSC osteogenic differentiation	^181^
DPSCs	DPSC+PRP	Increased DPSCs proliferation at 0.5% and 1% PRP concentration but decreased at 5% concentration. Long-term treatment with 1% PRP showed most significant enhancement at 96 h.	^182^
Human ASCs	culture medium containing 10 mL·L^−1^ PRP	Increased ASCs osteogenic differentiation and proliferation	^183^
Human MSCs	calcium phosphate scaffolds+PRP	Increased MSCs osteogenic differentiation and proliferation	^184^
Cartilage			
Human MSCs	10% PRP	Increased MSCs proliferation and chondrogenesis	^173^
Human BMSCs	10% PRP	Increased MSCs migration and proliferation	^185^
Human ASCs	10% PRP	Increased ASCs proliferation and preservation of immunophenotype and differentiation	^186^
Mouse MDSCs	10% PRP	Upregulation of type II collagen and improved MDSCs proliferation	^187^
Nude rat MDSCs	PRP+VEGF antagonist+BMP-4	Type II collagen increased	^188^
Sheep MDSCs	PRP	Increased MDSCs proliferation	^189^
Human ADSCs	PRP and insulin in 3D collagen scaffold	Increased chondrogenic and osteogenic differentiation of ASCs	^190^

Abbrevations: BMSC, bone marrow-derived mesenchymal stem cell; DPSC, dental pulp stem cell; MDSC, muscle-derived stem cell; MSC, mesenchymal stem cell; PRP, platelet-rich plasma; VEGF, vascular endothelial growth factor.

**Table 4:** PRP application in animal models

Cell type	Application	Animal model	Outcome	Reference
Rat BM-BMSCs	PRP gel/calcium phosphate particles	Rat femoral defect (2.5×5 mm)followed up at 4 weeks.	Increased BMSCs alp activity and proliferation	^207^
Equine MSCs	Gelatin/β-tricalcium phosphate (GT) sponge loaded with MSCs and BMP2	Equine osteochondral defects	The GT with MSCs/BMP2 group showed significantly higher macroscopic scores than the control group. In addition, hyaline cartilaginous tissue was detected in the test group in areas larger than those in the control group.	^208^
Murine ADSCs	(ADSCs) combined with PRP, and implanted on bone mineral matrix (BMM)	Subcutaneous mouse	The highest relative expression of bone-related genes and osteocalcin expression was found at the 15th day of *in vitro* osteogenic induction of the ADSCs. Expression peaks of bone-related genes in implants were at 2 and 4 weeks, but they significantly decreased at 8 weeks. The signs of resorption, formation of callus-like tissue positive for osteocalcin and increased presence of bone cells were found in experimental sites compared with control sites.	^209^
ASCs from inguinal fat pads of F344 inbred rats	ASCs with 5% PRP	Rat calvarial defect model	Increased osteogenesis and augmentative effects in the ASCs with 5% PRP group on bone regeneration *in vivo* as compared with the control.	^210^
Human PDLSCs	(PDLSC) sheets and 1% PRP	Subcutaneous immunocompromised mice	Based on the production of extracellular matrix proteins, the results of scanning electron microscopy and the expression of the osteogenic genes ALP, Runx2, Col-1 and OCN, the provision of 1% PRP for PDLSC sheets was the most effective PRP administration mode for cell sheet formation as compared to without PRP intervention.	^211^
Rabbit osteogenic-induced MSCs	Ceramic block with osteogenic-induced MSCs & PRP	Critical-sized segmental tibia defect in rabbits	The experimental group tibiae achieved the highest compressive strength (43.50±12.72 MPa) compared to those treated with control (23.28±6.14 MPa).	^212^
Rat UC-MSCs	(UC-MSCs) with 10% PRP	Rat critical-sized calvarial defects	PRP enhanced UC-MSC proliferation, and 10% PRP caused the strongest ALP and Alizarin red staining. At 7 days, the expression levels of ALP, Collagen 1 (COL-1) and Runt-related transcription factor 2 (RUNX2) in the PRP group were higher than those in the FBS group.	^213^
Rabbit ADSCs	ADSCs+PRP in alginate microspheres	*In vivo* subcutaneous injection of New Zealand rabbits	A blood vessel network was found within the 10% PRP and 15% PRP-ADSC implants, which was associated with a significant increase inmineralization.	^214^
BMSCs	PRP/BMSCs gel membrane	Ectopic mouse model and rabbit segmental bone defect model	The cells secreted significant amounts ofsoluble proangiogenic factors, such as PDGFBB, VEGF, and interleukin-8 (IL-8)	^84^
Murine MSCs	MSC associated with 50.0 μL of plasma gel containing 1.0×10^9^ autologous platelets	C57BL/6 gfpGFP(+) Mice and cranial defect of 6.0mm in diameter	At 10 days, the area of new bone formationwidened, at 30 days, was higher toward the center of the defect and at day 60, identified himself to the larger amount of bone tissue and more organized than had hitherto been observed	^215^
Canine DPSC, BMSC, and periosteal cells (PC)	DPSC, BMSC, and PC with PRP	Mandibular implants in dogs	DPSC showed the highest osteogenic potential around dental implants.	^216^
Angiopoietin 1 gene-transfected rabbit BMSCs	Angiopoietin 1 gene transfected BMSCs+PRP	Radial segmental bone defects (15 mm in length) were created in 20 3-month-old New Zealand rabbits	Callus formed at 4 weeks, partial bony union was observed at 8weeks, and complete union at 12 weeks.	^217^
Canine MSCs	A nanofiber scaffold, Pura Matrix (PM)+dMSCs+PRP	Implants in the region of first molar and all premolars in the mandibular regions of dogs	Bone-to-implant contact was highest in the PM, dMSCs, and platelet-rich plasma group at 55.64%.	^218^
Stem cells from deciduous teeth, dental pulp, and bone marrow	Stem cells from deciduous teeth (DTSCs), dental pulp (DPSCs), and bone marrow + PRP	Bone defects were prepared on both sides of the mandible canine mandible	Histologically, the cMSCs/PRP, cDPSCs/PRP, and cDTSCs/PRP groups had well-formed mature bone and neovascularization compared with the control (defect only) and PRP groups at 4 and 8 weeks, respectively, and the mineralized tissues in cMSCs/PRP, cDPSCs/PRP, and pDTSCs/PRP specimens were positive for osteocalcin at 8 weeks.	^219^
Human alveolar BMSCs (hABMSCs)	(hABMSCs)+0.5% PRF(platelet-rich fibrin)	Critical-sized defect in mice calvaria	Transplantation of the fresh PRF into the mouse calvarias enhanced regeneration of the critical-sized defect.	^220^
Dog MSCs (dMSCs)	dMSCs and/or PRP	Adult hybrid dog's mandible region	PM/dMSCs and PM/dMSCs/PRP groups showed a significant increase at all weeks compared with the control, PM, or PM/PRP in new bone formation.	^221^
MSCs	MSCs + PRP	Critical-size long-bone defects in diaphyseal rabbit model	PRP yielded better bone formation with CDHA scaffold as determined by both histology and micro-computer tomography (p<0.05) after 16 weeks.	^184^
MSCs	PRP	Sinus augmentation in minipigs	After 12 weeks, a significant increase in bone formation occurred in the Test sites compared with the control sites. In addition, BIC was significantly greater in the test sites compared with the control sites in the regenerated area	^222^
MSCs	Fibrin glue and PRP	Simultaneous implant placement and bone regeneration around dental implants in hybrid dogs	dMSCs/PRP/fibrin demonstrated the highest 53% bone implant contact at 8 weeks.	^223^
MDSCs	PRP	Osteoarthritis model for nude rats	Increase in type II collagen and decreased chondrocyte apoptosis.	^188^
MDSCs	PRP	New Zealand white rabbit(osteochondral defect)	Higher cartilage gene and protein expression. Improved immunohistochemical and histological characteristicsin association with the MDSCs with PRP group	^224^
Allogeneic ASCs	PRP	Equine superficial digital flexor tendonitis (SDFT)	At 24-month follow-up, reinjury rate was only 10.5%. Decreased pain.	^225^
Bone marrow aspirate concentrate	PRP	porcine osteochondral defect model	The Bone marrow aspirate concentrate group with PRP demonstrated increased collagen type II. Asignificant improvement of the histological characteristics.	^226^
Synovial membrane-derived MSCs	PRP	osteochondral defects in a new Zealandrabbit model	Increased type II collagen content, glycosaminoglycan content, cumulative histologic scores, and number of proliferating cells.	^227^
Autologous BMSCs	PRP	Equine tendonitis and desmitis	Implanted MSCs caused no adverse reactions and thirteen out of the 18 inoculated horses returned to race competitions. On the contrary, no improvement was seen in the twelve animals of group 2 treated with pin firing, that were not able to resume sport activity.	^228^
ASCs	Subcutaneous injection of the PRP alginate microspheres	Mouse subcutaneous	Blood vessel network was found within the 10% PRP and 15%PRP-ADSC implants. Significant increase in mineralization.	^214^
MSCs	PRP/deproteinized bone matrix (DPB)	1.5-cm segmental radial defects in New Zealand whiterabbits	The implantation of allogeneic PRP/(DPB) constructs group demonstrated successful bridging of the 1.5-cm segmental radial defects in rabbits, achieving similar healing capacity as autologous MSC/DPB constructs (MSC+DPB), with greater bone formation and vascularization than DPB alone, shown by histomorphometric analysis, bone mineral density measurement, and radionuclide bone imaging.	^229^
BMSCs	PRP	5 mm calvarial defects in New Zealand white rabbits	Substantial bone regeneration was observed at the calvarial defect restored with PRP incorporating the induced BMSCs at 8 weeks post implantation. In contrast, no bone regeneration was detected at the defects implanted with the whole blood incorporating BMSCs, whether the BMSCs were induced or not.	^199^
Rat BMSCs	PRP	Achilles tendon ruptures created surgically in Sprague Dawley rats	The use of rBMSC and PRP in the Achilles tendon ruptures when the tendon is in its weakest phase positively affected the recovery of the tendon in histopathologic, immunohistochemical, and biomechanical manners compared to the control group (*P*<0.05). The levels of pro-inflammatory cytokines TNF-α, IFNγ, and IL-1β were significantly low, the levels of anti-inflammatory cytokines and GFs playing key roles in tendon recovery, such as IL2, VEGF, TGF-β, and HGF, were significantly higher in the MSC group than those of the PRP and control groups (*P*<0.05).	^230^

Abbrevations: ADSC, adipose-derived stem cell; BMSC, bone marrow-derived mesenchymal stem cell; DPSC, dental pulp stem cell; IFNγ, interferon-γ; IL-1β, interleukin-1β; MDSC, muscle-derived stem cell; MSC, mesenchymal stem cell; PRP, platelet-rich plasma; TGF-β, transforming growth factor-β; TNF-α, tumor necrosis factor-α; VEGF, vascular endothelial growth factor.

**Table 5:** PRP application in the clinic

Sample size (patients)	Treatment	Time	Outcome	References
44	Open-wedge high tibia osteotomy (HTO) with or without Human MSC therapy and PRP injection.	24–36 months	The MSC-PRP group showed significantly greater improvements in the KOOS subscales for pain and symptoms. Arthroscopic evaluation, at plate removal, showed that partial or even fibrocartilage coverage was achieved in 50% of the MSC-PRP group patients but in only 10% of the patients in the PRP-only group (*P*<0.001).	^231^
24	Delayed fracture union: PRP, sorted Human MSCs in suspension and DBM (Ignite ICS injectable scaffold)	2, 6 weeks, 3, 6, 9 and 12 months.	No significant difference in VAS (visual analog scale), at 6-month time point, all fractures in the intervention group had healed, 25% of the control group experienced delayed union as their fractures did not unite by the 3-month follow-up.	^232^
72	Bone non-union: 6–8 mL platelet-rich plasma prepared by centrifugalizing venous blood and MSCs extracted from human umbilical cord.	2 years	No loosening and breakage of internal fixation were observed in two groups at 2 years. The motility and function of hip, knee and ankle were good. Significantly higher healing rate in MSC+PRP group.	^233^
6	Human MSC+PRP −972 269 (range 524 480–2 033 000) in sinus augmentation and alveolar ridge augmentation.	6 months	At 6 months after loading, as tested after removal of the prosthetic reconstruction, all implants maintained stability. Marginal bone resorption at 6 months after loading did not exceed 1.5 mm.	^234^
23	Injectable Human BMSCs and autologous PRP	3, 6 months and 1 year	Increased bone formation and Osseointegration.The mean regenerated bone height was 8.2±1.6 mm and 8.0±1.4 mm, and the average alveolar bone height was 15.6±1.2 mm and 15.1±1.4 mm, at 3 and 6 months, respectively. There were significant differences between pre-operative values and post-operative ones (at 3 and 6 months). No perforations of the Schneider membrane were found and the inserted implants were successful after 1 year.	^235^
01	Human MSCs and PRP	6 months	Re-examination at 6 months demonstrated that the application of MSCs-PRP gel at periodontal sites with angular defects, resulted in a 4-mm reduction in probing depths and a 4-mm clinical attachment gain, while bleeding and tooth mobility disappeared. Radiographic assessments showed that the bone defect had been reduced in depth.	^236^

Abbrevations: BMSC, bone marrow-derived mesenchymal stem cell; DBM, demineralized bone matrix; MSC, mesenchymal stem cell; PRP, platelet-rich plasma.
